# Reciprocal interferences of the left ventricular assist device and the aortic valve competence

**DOI:** 10.3389/fcvm.2022.1094796

**Published:** 2023-01-09

**Authors:** Olga Vriz, Ali Mushtaq, Abdullah Shaik, Ahmed El-Shaer, Khalid Feras, Abdalla Eltayeb, Hani Alsergnai, Naji Kholaif, Mosaad Al Hussein, Dimpna Albert-Brotons, Andre Rudiger Simon, Felix Wang Tsai

**Affiliations:** ^1^Heart Centre Department, King Faisal Specialist Hospital and Research Center, Riyadh, Saudi Arabia; ^2^School of Medicine, Alfaisal University, Riyadh, Saudi Arabia

**Keywords:** heart transplant, left ventricular assist device (LVAD), aortic regurgitation, transcatheter aortic valve implantation (TAVI), surgical intervention

## Abstract

Patients suffering from end-stage heart failure tend to have high mortality rates. With growing numbers of patients progressing into severe heart failure, the shortage of available donors is a growing concern, with less than 10% of patients undergoing cardiac transplantation (CTx). Fortunately, the use of left ventricular assist devices (LVADs), a variant of mechanical circulatory support has been on the rise in recent years. The expansion of LVADs has led them to be incorporated into a variety of clinical settings, based on the goals of therapy for patients ailing from heart failure. However, with an increase in the use of LVADs, there are a host of complications that arise with it. One such complication is the development and progression of aortic regurgitation (AR) which is noted to adversely influence patient outcomes and compromise pump benefits leading to increased morbidity and mortality. The underlying mechanisms are likely multifactorial and involve the aortic root-aortic valve (AV) complex, as well as the LVAD device, patient, and other factors, all of them alter the physiological mechanics of the heart resulting in AV dysfunction. Thus, it is imperative to screen patients before LVAD implantation for AR, as moderate or greater AR requires a concurrent intervention at the time of LVADs implantation. No current strict guidelines were identified in the literature search on how to actively manage and limit the development and/or progression of AR, due to the limited information. However, some recommendations include medical management by targeting fluid overload and arterial blood pressure, along with adjusting the settings of the LVADs device itself. Surgical interventions are to be considered depending on patient factors, goals of care, and the underlying pathology. These interventions include the closure of the AV, replacement of the valve, and percutaneous approach *via* percutaneous occluding device or transcatheter aortic valve implantation. In the present review, we describe the interaction between AV and LVAD placement, in terms of patient management and prognosis. Also it is provided a comprehensive echocardiographic strategy for the precise assessment of AV regurgitation severity.

## Introduction

Aortic valve pathophysiology and the associated risk factors predisposing to AR development in patients with left ventricular assist device (LVAD) should be taken into account considering the increasing number of patients with end-stage heart failure (HF) that temporarily or permanently are implanted, despite being on optimal medical management (1) while waiting for cardiac transplantation (CTx). This is particularly important since CTx is vital for enhancing life expectancy, quality of life (QoL), and functional status (2, 3), however, organ donor shortage is an ongoing obstacle and, as a result, fewer than 10% of patients with severe, refractory HF receive CTx (4, 5). An alternative for this patient cohort is mechanical circulatory support with LVAD (6, 7). LVADs work by unloading the left ventricle (LV) and providing appropriate pressure, thus increasing cardiac output. The subsequent structural and functional changes improve survival and QoL for end-stage HF patients, despite being tethered to an external power source (7–10). However, long-term LVAD placement has side effects, related to the device, heart function, or cardiac valves, including the aortic valve (AV). Co-existing cardiac valve dysfunction can make LVAD implantation and effectiveness more challenging. Multiple factors can be responsible for AV malfunction, compromising the benefits of the device (11). Aortic regurgitation (AR) affects at least 25–30% of patients within the first year of implantation, which can lead to poor pump efficiency, worsening HF, and increased mortality (12, 13). However, there is no agreement on the prognostic role of AR in LVAD patients in terms of outcomes (14–18) and therapeutic management (19–21).

In the present review, we describe the interaction between AV and LVAD placement, in terms of patient management and prognosis. We also provide a comprehensive echocardiographic strategy for the precise assessment of AV regurgitation.

### Left ventricular assist device

**FIGURE 1:**
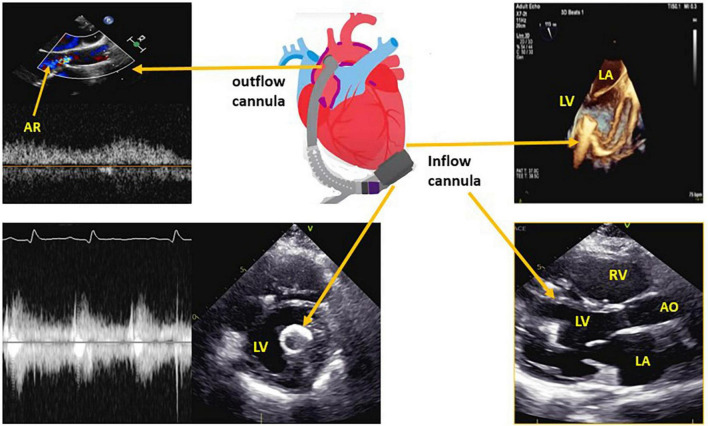
Left ventricular assist device (LVAD) structure with the corresponding echocardiographic images for the inflow cannula and outflow cannula. LV, left ventricle; LA, left atrium; RV, right ventricle; AR, aortic regurgitation.

The function of the LVAD is to reduce the workload of the LV by pumping blood *via* a LV apical cannula to the aorta, to maintain systemic perfusion (Figure 1).

Left ventricular assist devices can be divided into pulsatile or continuous flow. Pulsatile flow LVADs (PF-LVAD), mimic the heart’s natural rhythmic motion (e.g., Berlin heart), whereas continuous flow LVADs (CFL-VAD) use a motor operating at a fixed speed, resulting in continuous ejection of blood into the aorta. This causes a decreased pulse pressure and a non-palpable peripheral pulse. CF-LVAD can be further subdivided into axial flow (HeartMate II, Jarvik 2000, and DeBakey) and centrifugal flow (VentrAssist, HeartWare, Levacor, and HearMate III). Axial flow employs a turbine rotation, pushing blood into the outflow cannula, whereas centrifugal flow draws blood along the rotor’s axis and propels it tangentially into the systemic circulation. Continuous flow LVAD has substantially prolonged the longevity of the pump (22).

Between 2011 and 2022, there has been a progressive increase in LVAD implantation (26,688 devices placed), with a drop in 2020, because of the COVID-19 pandemic effect on cardiac surgical volumes in the United States (23). At present, HeartMate III (Abbott) is the most employed LVAD in clinical practice (24).

Left ventricular assist device is employed in a variety of clinical settings, based on the goals of therapy:

a) Short term: Bridge to recovery. These are patients awaiting cardiac surgery, or who suffer from ventricular arrhythmias, cardiogenic shock, or heart failure that are refractory to medical management.
b) Medium term: Bridge to Transplantation (BTT) and Bridge To candidacy (BTC). These patients require additional support while awaiting CTx or are currently unfit for CTx, but not have absolute contraindications (23, 25–27). BTC patients have improved utilization rates, increasing from 26.9% (2011–2015) to 32.5% in (2015–2020) (23).
c) Long-term: Destination Therapy (DT). Patients who are ineligible for CTx due to age, comorbidities, or psychosocial factors can have prolonged survival with LVAD implantation. Due to the new heart allocation, heart strategy by the United Network for Organ Sharing (UNOS) in 2018, DT has increased from 42.7% (2011–2015) to 50.4% (2015–2020) (28). Reported survival rates for DT are 82.8 and 74.1% at 1 and 2 years, respectively (23). LVAD implantation is also used as a DT in facilities that lack CTx facilities (29).

Left ventricular assist device implantation is associated with several complications (Table 1). Despite the complication rate, LVAD use has shown a significant reduction in morbidity rate from any cause, as compared with medical treatment, as demonstrated by the REMATCH trial (6). The 2-year survival rates for patients using the latest CF-LVAD (HeartMate III) are equivalent to those following CTx, with survival rates of 80 and 70% at 1- and 2-years, respectively (30, 31). An overall survival rate of 74.5% was seen when fully magnetized centrifugal-flow LVADs were used in real-world population using 2-year results from the ELEVATE registry (32). The MOMENTUM 3 trial demonstrated that, in centrifugal-flow LVADs, the 2-year survival rate was 84.5%, while the stroke-free and need for reoperation rate, due to LVAD malfunction, was 76.9% (24), confirmed by the more recent MOMENTUM 3, 5 years outcome (33). Additionally, even though numerous patients have been planned to be implanted as DT or BTT (mid and long term destination), some of these can turn in bridge to recovery when weaning criteria are satisfy, included no AR or maximum grade I AR. In these selected patients with HF secondary to chronic cardiomyopathy who underwent LVAD removal for complete or even incomplete cardiac recovery, had a 66% freedom from HF and 10.6% mortality after 5 year post-weaning (34).

**TABLE 1:** Left ventricular assist device (LVAD) complications.

Specific complication (106)
Suction event (reduced pre-load), the inflow cannula is positioned incorrectly, or the left ventricular chamber is excessively decompressed (LVAD speed is too high).
Pump thrombosis (more common in continuous LVAD) originate in the pump or the inflow or outflow cannula.
**Mechanical failure.**
**Associated complications**
**Bleeding** (107–109): From several sources. GI bleeding affects 15–30%, more commonly seen in continuous flow LVAD. Platelet aggregation is impaired by shear stress from the LVAD impeller, which might result in acquired von Willebrand’s syndrome along with reduced pulsatility increases bleeding risk.
**Cerebrovascular complications** (110–112): Hemorrhagic and ischemic, highest risk first year after implantation. Infection when on a LVAD raises the likelihood of a hemorrhagic stroke. Women have increased risk vs. men.
**Infection:** Device-related, device-specific, non-LVAD (rate > 42% in the first-year post-implant).
**Right ventricular failure** (113, 114): Major cause of morbidity and mortality, in 15–40% of patients.
**Dysrhythmia** (115–118): Severe dysrhythmias can be tolerated if LVAD produced adequate cardiac output, but RV function can be compromised. Often arise secondary to ischemia, scar tissue or irritation of myocardial wall by the cannula.
**AR** (20, 21, 119): Up to 10% within 6 months, 25 and 50% at 12 and 18 months, respectively. More common in patients with closed aortic valve, long term support, pre-existing AR often due to leaflet remodeling from exposure to high LVAD flows.

LVAD, left ventricular assist device; RV, right ventricle; AR, aortic regurgitation.

### Left ventricular assist device and AR

Risk factors for progression of AR in LVAD patients include longer duration of LVAD placement, use of CF-LVAD, DT, older age, female gender, smaller BMI, and mild pre-implant AR (12–14, 21, 35, 36). Short-term data suggests that 10–55% of patients will develop *de novo* AR over the first 6 months post-implantation (14, 36). This also seems to hold true in patients implanted as DT (18). At 2.5-year follow-up, 43.2% of those patients with mild AR pre-LVAD developed moderate to severe AR (18). Moreover, it was reported that at 2 years follow-up, 33% of the LVAD patients developed more than moderate AR and only 30% of AVs opened at least partially or intermittently.

#### AR mortality and morbidity

**FIGURE 2:**
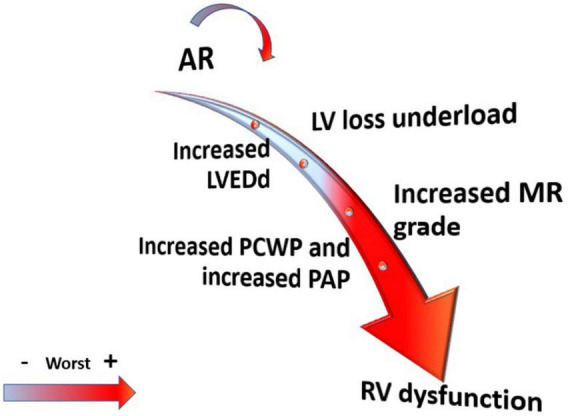
Effect of aortic regurgitation (AR) severity on left ventricle (LV) and right ventricle (RV) structure and hemodynamic.

There is no uniform consensus on mortality and morbidity on the effect of AR in LVAD patients. Although many studies have shown a progressive increase in *de novo* and progression of AR over time (16), most of them show no difference in survival between patients with AR compared to those who did not develop AR (13, 16–18, 36, 37). For example, Holly et al. (16) reported that 15.2% of patients developed severe AR out of 237 implanted with CF-LVAD but no difference in survival was noted when patients who developed AR were compared with those who did not develop AR. Cowger et al. (13) found that the development of AR is a common phenomenon and did not affect mortality except in those with pre-LVAD implantation significant right ventricle (RV) dysfunction. In general the hypothesis of those that support no direct relation between AR and mortality, is that patients with AR are more likely to have more complications including mitral and tricuspid regurgitation, hemolysis, worse RV function at long-term follow-up, higher hospital readmissions for HF, and more probable to remain in NYHA class III (13, 18, 21, 37–39) (Figure 2).

On the other hand, the INTERMACS study (14) found that patients on LVAD with significant AR had increased mortality compared to those with no AR (49.1% vs. 36.5% at 5-year, *p* < 0.001) after adjustment for confounders. Moreover, patients with severe AR had at least moderate mitral regurgitation (MR), leading to lower systolic blood pressures and cardiac output, and higher rehospitalization rates for HF. Auvil et al. (40), in their cohort of patients, found that moderate AR was an independent predictor of 2-year mortality after LVAD implantation and there was a significant difference in survival among patients with no AR, mild and moderate AR. On the other hand, they did not find any difference among AR groups in terms of 6-min walking test and prevalence of RV failure. In Table 2 are summarized all the studies we found on Pubmed using keywords as LVAD, AR and mortality/survival, excluding case reports (13–18, 36–48).

**TABLE 2:** Literature review of multiple studies studying the association between left ventricular assist device (LVAD) and AR.

References	Year of publication (year of study)	Device under study	Number of patients	Mean age of patients (years)	Median follow up	Bridge to transplant (%)	Destination therapy (%)	Pre-operative AR (%)	Post-LVAD AR (*de novo*) (%)	Effect of AR on survival/mortality
Cowger et al. (13)	2014 (2000–2011)	HeartMate II	166	56.6 ± 12	461 days	79	21	94% with mild or less	78.3% with < moderate AR 21.7% with > moderate AR	Similar survival between different AR groups ֏
Gasparovic et al. (37)	2022	Multiple	396	53 ± 12	511 days	68	18	0 (none)	39% experienced progression of AR	No difference between AR and non-AR patients ֏
Toda et al. (47)	2011 (1999–2009)	Multiple	47	35 ± 11	1,098 days	NA	NA	0 (none)	38% mild AR	Survival significantly worse in *de novo* AR
Auvil et al. (40)	2020 (2008–2018)	HeartMate II and HeartWare	221	57	547 days	NA	NA	4.5% moderate AE 40.3% mild AR	36% mild AR at 6 months 4% moderate AR at 6 months	Statistically significant increase mortality in moderate AR group
Tanaka et al. (18)	2020 (2006–2018)	HeartMate II and HeartWare	604	59.6 ± 11 mild AR 54.7 ± 12 no AR	NA	55.7%	44.4%	18.4% mild 81.6% trace or none	NA	Survival similar between AR and no AR group ֏
Saeed et al. (43)	2016	HeartWare	34	57 ± 12	408	68%	NA	6% mild AR	24% with trace/mild AR 3% moderate AR	No difference in survival between patients with AR versus no AR
Holly et al. (16)	2016 (2005–2013)	HeartMate II	210	63 ± 13 Moderate or severe AR 55 ± 14 No moderate or severe AR	582 days	79.5%	NA	NA	15.2% moderate or severe AR	No difference in survival between AR and non-AR patients
Da Rocha e Silva et al. (41)	2015 (2009–2013)	HeartWare and HeartMate II	102	54 ± 12 overall 53 ± 12 no AR 56 ± 13 > mild AR	572 days	NA	NA	NA	69% no significant AR 31.4% moderate to severe AR	NA
Park et al. (42)	2022 (2007–2017)	Multiple	219	61.5	602 days	43.4%	57.5%	39.7%	29.7% with moderate- severe AR	Significant increase in mortality and morbidity in patients who developed AR
Patil et al. (17)	2014 (2006–2012)	HeartMate II and HeartWare	93	39.9 ± 14.1	527 days	NA	NA	Difficult to determine	14% with moderate and 2.1% with severe AR	No difference in survival between AR and non-AR group
Pak et al. (36)	2010 (2004–2009)	HeartMate II and HeartMate I	63 HeartMate II 67 Heartmate I	53.2 ± 13.9 HMI 55.5 ± 13.0 HMII	134 days HMI 204 days HMII	81% HMI 84%HMII	19.4% HMI 15.9% HMII	NA	6% in HMI 14.3% in HMII	NA
Naganuma et al. (38)	2021 (2008–2017)	Multiple	53	43.9 ± 14.1	856.3 days	NA	NA	3.8%	17% with moderate to severe AR	No difference in mortality between patients with AR ≥ grade 3 compared to those with AR < grade 3
Hiroaka et al. (44)	2015 (1997–2012)	Multiple	99	58.3 ± 12.3	314 days	NA	46%	17.1% mild AR	52% AR	No difference in mortality between *de novo* AR and non-AR group ֏
Soleimani et al. (46)	2012 (2008–2010)	HeartMate II and HeartWare	66	NA	374.5 days	54.5%	41%	NA	9.5% AR	NA
Bhagra et al. (48)	2016 (2009–2013)	HVAD	71	47 ± 12.6	624 days	100%	NA	4.1% mild AR	24.5% > mild AR 1 year support 28% > mild AR 3 years support	NA
Kagawa et al. (15)	2020 (2004–2018)	Multiple	316	59.5 ± 2.24	469 days	42.9%	57.14%	36.2% with mild AR	13.3% AR	Higher morality in patients with significant AR
Truby et al. (14)	2019 (2006–2016)	Multiple	10,603	NA	13.4 months mean	57%	42.2%	30.6% mild AR	13.2% mod to severe AR	36.5% survival after 5 years ψ
Aggarwal et al. (45)	2013 (2005–2011)	Heartmate II	79	63.2 ± 11.8	761 days	13%	87.3%	6.3% trivial	52% with mild or greater AR	40% survival after 5 years ֏
Imamura et al. (39)	2015 (2006–2013)	Multiple	52	41 ± 13	NA	100%	NA	0 (none)	21%	92% survival after 2 years ֏

#### Different LVAD modalities and their effect on AV function

Studies have been performed to assess the relationship between different LVAD devices and AR (49–51), and, as previously mentioned, AR is a determinant in morbidity and mortality which are described in detail in Table 2. As compared to PF-LVAD, CF-LVADs provide an overall larger reduction in LV end-diastolic pressure and volume but also cause derangements of the pressure-volume loop. CF-LVADs also improve LV systolic flow, aortic flow, LV systolic pressure, and mean arterial pressure. However, PF-LVADs have lower rates of developing more advanced AR than CF-LVAD patients (15, 35, 36, 52). Park et al. (42) described that patients with CF-LVAD were two times more likely to develop AR than patients on PF-LVAD support. Hatano et al. (52) considered AR frequency among multiple device brands, either CF-LVAD or PF-LVAD, and found that CF-LVAD was an independent risk factor for the development of AR. Kagawa et al. (15) reported that 30% of their 316 patients, developed AR at 1 year from CF-LVAD implantation. The effect of the different LVAD was explained by Imamura et al. (53). According to the authors, PF-LVAD unloads the LV only during the diastolic time, whereas CF-LVAD unloads the LV throughout the all cardiac cycle with a constant transvalvular pressure less than 0 mmHg that avoid AV opening. The time of AV opening was higher, AR rate lower, LV diameter in diastole lower and higher LV ejection fraction in PF-LVAD patients compare to CF-LVAD. It seems that CF-LVAD *per se* has a LV remodeling effect that ultimately contributes to the development of AR. Moreover, complete LV unloading is associated with higher myocardial fibrosis and cardiac stiffness with decreased coronary flow which promotes inflammation and myocardial fibrosis (54–56).

Although pulsatility may have some advantages, the improvements in size, reliability, efficacy, and durability of the current generation of CF-LVADs have made them the LVAD of choice. The latest generation of CF-LVAD allow some intermittent aortic valve opening for washing of the aortic root. HeartMate 3 LVADs have demonstrated a certain degree of pulse pressure in animal models. Actually it represents 80–90% of all implants since the U.S. FDA approval in October 2018 (57).

However, AR, as said, develops over time in CF-rotatory-LVAD pump and the recirculation of blood flow is more severe in CF than PF system with more severe reduction in blood flow to the end organs and signs of HF, despite the increase of LVAD speed. For these reasons, AR more than mild should be addressed before or during support device implantation (58).

Newer LVAD’s have built-in artificial pulsatile, but their impact on the AV remains to be established (CorWave has been awarded the Medtech Award 2021) (59).

### AV pathophysiology and risk factors predisposing to AR development

The altered physiological mechanics of the heart, resulting from LVAD-host integration might cause the development of AV dysfunction (60).

#### Aortic valve-aortic root complex

Under normal conditions, the aortic root-aortic valve complex guarantees unidirectional, intermittent blood flow under optimal conditions, with laminar flow, minimal shear stress and resistance, and complete diastolic coaptation of the AV leaflets (61). Moreover, the AV endothelium has important regulatory functions, acting as a paracrine structure releasing regulatory cytokines (including tumor necrosis factor-α), anti-thrombotic and vasoactive factors (nitric oxide). The AV endothelium is also side-specific, sensitive to local flow and shear stress, with the aortic side exposed to higher flow, pressure, and turbulence than the ventricular side (62).

#### Device factors that predispose to the development of AR

The high shear stress produced by CF-LVADs is found throughout the whole cardiac cycle, exposing the aortic valve leaflets and aortic wall to continuous turbulent flow and higher arterial pressures. Normally, the valve leaflets relax in the open position when stretch and stress are low, allowing nutrient flow to the tissue. With CF-LVADs, the leaflets are usually persistently closed, so that the valve leaflets are under maximum stretch with a continuously high transvalvular gradient. This provides a stimulus for collagen production and increased inflammation, causing adverse remodeling of the valve leaflets, with retraction, degenerative involvement, and focal nodular calcification (20, 35, 63). In contrast to what naturally occurs in AV disease, focal lesions occur on the ventricular side of the AV as a consequence of valve dysregulation due to constant high transvalvular pressure (60). Post-transplant autoptic evaluation of LVAD hearts, revealed extensive tissue remodeling of the AV, in particular commissure fusion in 71–88% of patients (64, 65). In addition, it is also postulated that, as a consequence of the closed aortic valve cusps and blood stasis on the ventricular side, thrombus formation is more likely (66), along with forming a commissural fusion and accelerating degenerative processes of the AV (60, 67, 68).

Shear stress can also affect the aortic wall, as the outflow graft can be smaller than the native aorta, with higher velocity and flow. The consequence of this process is aortic dilatation, with progressive thinning of the aorta media layer, decrease in smooth muscle cells and elastic fibers of the media layer (69–71). Finally, the implantation position of the outflow anastomosis is also important, suggested to be 2 cm above the sino-tubular junction and more than 90 degree inclination, to reduce blood flow stagnation in the aortic root and thrombi formation with normal wall shear stress and moderate local pressure values (72). If the cannula is too far away from the AV, in the ascending aorta or descending aorta, the reduced blood washout near the AV can promote thrombus formation (73). On the other hand, if the outflow cannula is attached too close to the AV, can be responsible of the AV cusps distortion and mal-coaptation due to increased local pressure, diastolic transvalvular pressure gradient promoting AR (74, 75).

Absence of AV opening, distortion of AV cusps and dilatation of the aortic root are factors that predispose to *de novo* AR and worsening of pre-existing AR. This pathophysiology is seen in animal models, leading to aortic atrophy and worsening AR (69).

#### Patient factors that predispose to the development of AR

In addition to the factors related to the interaction LVAD-host, there are also patient factors that predispose to developing AR. In the metanalysis of Deo et al. (76), that considered 7 observational studies (657 patients), 65% of the them used CFLVAD, it was found that pre-operative parameters affecting the development of AR were older age, female sex, and low body surface area. Mitral regurgitation was also independently related to the development of AR. Post-LVAD implantation factors that played a role in the development of AR were larger aortic root and aortic sinus diameter, closed aortic valve and longer duration of support (15, 16, 76).

## Assessment of AR using echocardiography and patient selection

According to European Society of Cardiology (ESC)/American Society of Echocardiography (ASE) guidelines (77), it is crucial to evaluate the structure and functions of cardiac muscle and valves, with a keen focus on AV, using transthoracic (TTE) and/or transesophageal (TEE) echocardiography before LVAD implantation, during implantation and follow-up. Echocardiography, either TTE or TEE, are sensitive enough to detect valve anatomy and function. In Table 3 are summarized the measurements that have to be taken to select the patient for implantation and eventually plan other cardiac procedures (i.e., ASD closure). Table 4 reports the specific measurements for AR stratification while Table 5 illustrates the timing and parameters to be evaluated during implantation, post-procedure, and during follow-up (12, 20).

**TABLE 3:** Echocardiographic parameters pre-left ventricular assist device (LVAD) implantation (80, 120).

Parameters	Independent predictors of prognosis
LV dimension and function	LV volume > 120 ml/m2
RV function and dimension	TAPSE < 14 mm, RVS‘ < 10.8 cm/s, RVE‘ < 8.9 cm/s. FAC reaches < 20% the incidence of post-operative RV failure dramatically increases. Free wall longitudinal strain < −9%
Estimated PASP	PASP > 45 mmHg
MV Doppler	E/E‘ > 15, restricted filling or pseudo normal filling. DT < 140 ms
**Valve pathology**	
AS	Little significance on LVAD patients
AR	More than mild AR need to be addressed
TR	Severe TR > 2.5 m/s
Intracardiac communication	Intracardiac shunts (PFO, ASD, VSD): level, direction, and amount of shunt
Aorta dimensions	AV annulus, sinus of Valsalva, aortic root
Intracavitary clots	Yes/no. Echo contrast (Optison) can help in visualization

LV, Left ventricle; RV, right ventricle; PASP, pulmonary artery systolic pressure; MV, mitral valve; AS, aortic stenosis; AR, aortic regurgitation; TR, tricuspid regurgitation; PFO, patent forame ovale; ASD, atrial septal defect; VSD, ventricular septal defect; FAC, fractional area change; TAPSE, tricuspid annular plain systolic excursion; DT, deceleration time.

**TABLE 4:** Aortic regurgitation (AR) evaluation pre-left ventricular assist device (LVAD) implantation (78, 121–123).

The presence of mild to moderate AR is defined by	
Pressure half time (PHT)	<500ms
Vena contracta (VC) width	>0.3 cm
Jet-width/left ventricular outflow tract (LVOT) ratio	>25%
PISA Method	Regurgitant flow rate [2π × r^2^ × V_*Aliasing*_] (r is radius of the flow convergence in early diastole, and V_Aliasing_ is the Nesquit limit velocity at 0.35 cm/s). EROA = flow rate/peak aortic regurgitation rate in early diastole (CW). RVol = EROA × VTI of the aortic regurgitation (CW).
AR regurgitant volume (RVol)	RVol from RVOT (RVol = CO_*LVOT*_–CO _*RVOT*_). RVol from mitral valve (MV) (RVol = CO_*LVOT*_–CO_*MV*_).
RVol: More than 30 ml/beat in more than mild AR, more than 60 ml/beat in severe AR.	
Flow reversal in the transverse arch and/or descending/abdominal aorta with pulse-wave Doppler	Holo-diastolic flow is present in severe AR but not in moderate.
**Limitations**	
PHT	Affected by changes in LV and aortic diastolic pressures. Elevated LV end-diastolic pressures can reduce gradients driving AR, leading to an underestimation of AR severity on echocardiography (e.g., anesthesia, shock).
Eccentric AR jet	Difficult Doppler alignment and underestimation of PHT. Can be compensated by VC measurement, relation to LVOT diameter, holo-diastolic flow in abdominal aorta.
PISA method	Is not feasible in a significant percentage of patients due to interposition of valve tissue and difficulty in correctly identifying the flow convergence zone.
RVol quantification	From RVOT: Not always well visualized and difficult to measure accurately the RVOT diameter. No MR involvement. From MV: not done if more than mild MR is present. Difficulty in measuring accurately the mitral annulus.
Flow quantification techniques	Difficult in the presence of mitral regurgitation.

PHT, pressure half time; LVOT, left ventricular outflow tract; MR, mitral regurgitation; RVOT, right ventricular outflow tract; VTI, velocity time integral; CW, continuous wave; EROA, effective regurgitant orifice area; AR, aortic regurgitation; RVol, regurgitant volume; VC, vena contracta; CO, cardiac output; LV, left ventricle; PISA, proximal isovelocity surface area.

**TABLE 5:** Intraoperative evaluation and post-left ventricular assist device (LVAD) (77).

TEE intraoperative evaluation
***Global bi-ventricle evaluation:***
RV size and function (if LVAD alone)
LV unloading
Valvular abnormalities
Intracardiac shunts: level, direction, and amount of shunt
***Inflow and outflow cannula position and Doppler:***
*LV inflow cannula* in LV apex:
Parallel to septum and aligned with mitral inflow Unidirectional flow from left ventricle into the cannula
*LV outflow cannula* in ascending aorta:
Cannula position before and after chest closure
***Deairing during the following phases of surgery:***
From cannula placement until release of aortic cross-clamp From release of aortic cross-clamp to the end of cardiopulmonary bypass From the termination of cardiopulmonary bypass to the end of the operation
**Intra-operative and post-LVAD AV evaluation (TEE or TTE)**
*AV opening:* duration of cusp separation (from no opening to intermittent) depending on the degree of LVAD support (pump speed)
*AR:* can be intermittent, during diastole, almost continuous (from diastole to part of systole) or continuous (holosystolic and holodiastolic). See Table 4 for classical parameters for severity stratification.
**Additional AV measurement** (61, 81, 83)
Diastolic acceleration time (diastolic slope from the onset to the end of diastole) (>49 cm/s)
S/D ratio (is calculated by dividing the peak systolic velocity by the end diastolic peak velocity) (<5)
(these values correspond to at least moderate AR)
AR severity using PISA method considering temporal resolution: 2π (PISA)^^2^ × aliasing velocity × duration AR × HR

LVAD, Left ventricular assist device; LV, left ventricle; AV, aortic valve; AR, aortic regurgitation; VC, vena contracta; LVOT, left ventricular outflow tract; CW, continuous wave; HR, heart rate; PISA, proximal isovelocity surface area.

### Pre-implantation assessment

Stratification of AR severity pre-LVAD implantation is fundamental for patient management and should be done according to guidelines for accurate long-term prognosis since some of the parameters are predictors of future development of AR (78–80). Table 3 summarizes all the TTE parameters that should be considered and reported during the pre-implantation assessment. Patients with moderate to severe AR require surgical intervention (repair or replacement of the aortic valve). It is always important to remember in AR that the pressure difference between the two chambers (LV and aorta), the diastolic characteristic of the LV, and the LV volume all have an impact on the regurgitant flow. If the AR assessment by TEE is performed under sedation or general anesthesia, it is possible that there is a high LV diastolic pressure, low systemic resistances, and a small pressure gradient across the LV-aorta, which underestimates the degree of AR and can be avoided by increasing the blood pressure. As previously stated, sinus of Valsalva, sinus tubular junction and ascending aortic diameter should be documented since they are predictors of future AR development (Tables 3, 4).

### Peri-implantation assessment

Table 5 lists the variables to be verified during the procedure and the timing of the evaluation. During LVAD implantation transesophageal echocardiography (TEE) will be used either for the bi-ventricular function evaluation or AV function or correct alignment of the inflow-outflow cannula. The increased severity of AR following LVAD implantation necessitates a referral to the surgeon. The reason might be underestimation of AR during preimplantation or high suction of the inflow cannula (high pump speed). In terms of AV evaluation, it is critical to determine if AV opens, quantifying the opening (intermittent, continuous, by M-Mode), and the severity of AR if present. The optimal pump speed is the one that allows for at least intermittent AV opening (77).

### AR assessment under LVAD

**FIGURE 3:**
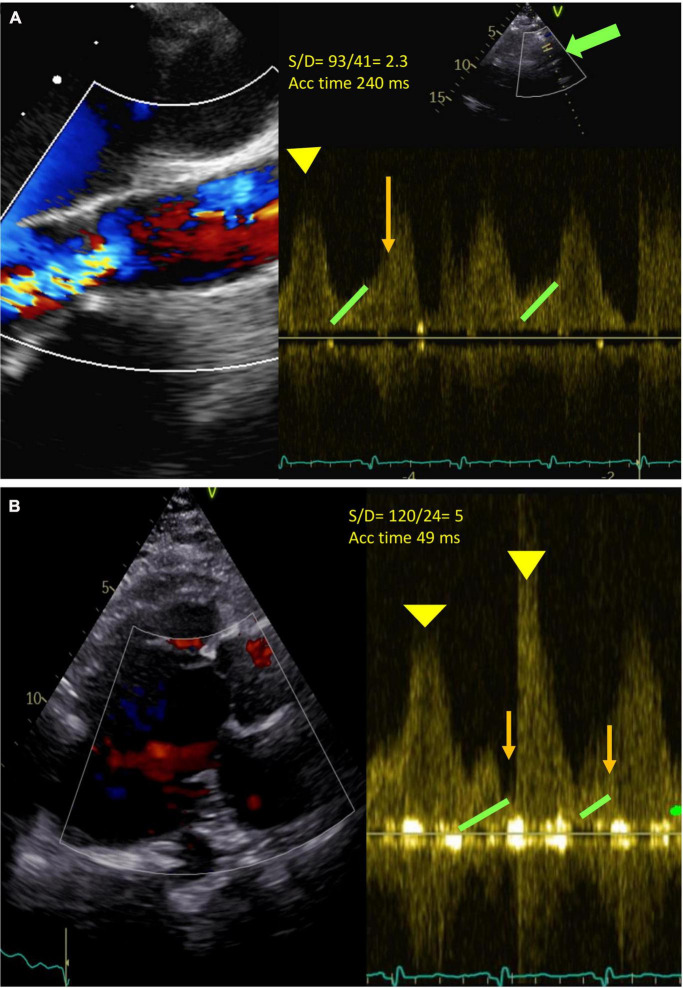
**(A)** Severe continuous (holo-systolic and holo-diastolic) aortic regurgitation (AR). Green arrow: outflow cannula. Pulse Doppler (PW) at about 1 cm from the outflow cannula (bold green arrow). Diastolic acceleration time 49 ms (orange line), S/D ratio 93/41 = 2.3 (<5 significant AR) (Yellow head arrow: systolic wave; green head arrow: end Diastolic velocity). Acceleration time 240 ms. **(B)** No AR. S/D 53/10 = 5. Acceleration time 49.

Traditional TTE tends to underestimate the severity of AR as it occurs throughout the cardiac cycle due to insufficient residual LV contractile forces to oppose the backflow in systole, thus even a relatively small AV orifice can account for severe AR. Aortic flow, as a result, becomes highly dependent on global hemodynamics such as LV pre-load, residual contractility, and heart rate (77). Furthermore, the quantitative parameters (pressure half time, vena contracta, proximal iso-velocity surface area) are unreliable in this context since the determinant factor to the assessment of regurgitant severity in a CF-LVAD patient is the measure of flow over time. In addition to the traditional parameters, other specific measurements are suggested to improve AR severity stratification such as those reported in Table 5. For example, pulsed wave (PW) Doppler of the outflow cannula is important for documenting laminar, unidirectional, low-peak velocity flows, and no regurgitation. The PW sample volume is placed at least 1 cm from the anastomosis (Table 5) and a pulsatile flow pattern is characterized by phasic changes in flow throughout the cardiac cycle, reaching the maximum during systole and minimum during diastole. This signal can be used to calculate two parameters: (1) peak systolic-to-diastolic (S/D) velocity ratio, which correlates negatively with AR, and (2) diastolic acceleration time, which correlates positively with AR (Figure 3) (81, 82). Diastolic acceleration time is the diastolic slope from the onset to the end of diastole and the S/D ratio is calculated by dividing the peak systolic velocity by the end diastolic peak velocity. An S/D ratio less than 5.0 and/or a diastolic acceleration time greater than 49.0 cm/s corresponds to at least moderate AR, defined as a regurgitant fraction > 30%. The relationship between diastolic flow on the outflow cannula and AR is self-evident: as AR worsens, diastolic flow through the outflow cannula increases (81). However, in our experience, it is not always easy to be accurate since the outflow cannula might not be seen and/or the PW sample is not aligned with the flow of the cannula.

As previously mentioned, the duration of AR should be considered when the AR is quantified (measure of flow over time). For this reason, the calculation of regurgitant volume (RVol) can be estimated by measuring PISA by M-mode, AR by CW, and the duration of AR (ms) adjusted by heart rate (83) (Table 5).

Importantly, the degree of AV opening can be significantly reduced or intermittent, depending on the LVAD speed. Ideally, LVAD support aims to open the aortic valve every two or three beats. M-Mode of the AV long-axis can help to quantify the degree of AV opening (intermittent opening, in which part of the cardiac cycle, extend AV opening) and if color is added also the AR characteristics (extent of AR into systolic period -electrical and mechanical-) It is suggested to acquire at low speed (25 mm/s) and at least 3–5 cardiac cycles.

### Follow-up after LVAD implantation

**FIGURE 4:**
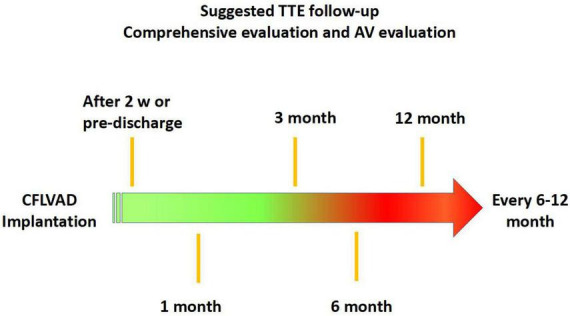
Flow chart follow-up after left ventricular assist device (LVAD) implantation.

Following LVAD implantation, it is recommended to perform routine TTE according to the algorithm shown in the flow chart (Figure 4).

**FIGURE 5:**
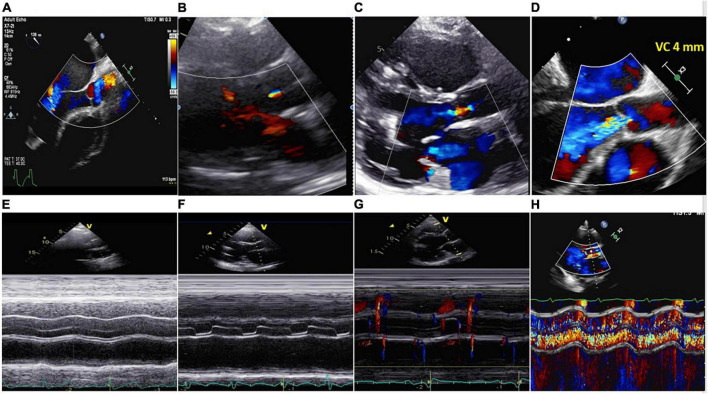
Upper part: **(A)** No aortic regurgitation (AR); **(B)** trivial AR; **(C)** mild AR; **(D)** severe continuous AR (VC 4 mm). Lower part: **(E)** Closed AV; **(F)** aortic valve open in end diastole and systole. No AR; **(G)** aortic valve opened in systole. No AR; **(H)** same patient of panel **(D)**. M-Mode showing severe systolic-diastolic AR. The valve was closed during the cardiac cycle.

Standard comprehensive echocardiography will be performed, with a particular emphasis on LV and RV dimensions, systolic function, inflow and outflow cannula interrogation, valvular apparatus included AV function (AR or aortic stenosis, AV normally open or closure time and its relationship with the cardiac cycle) (Figure 5). It is recommended to record 3–5 cycles. TTE should be performed according to the guidelines and remain consistent during the follow-up (77, 78). Since the quantification of AR in LVAD patients is quite challenging, a multiparametric approach is mandatory.

## How to manage AR or prevent *de novo* AR in LVAD patients

The consequence of AR determines backward flow through the AV during diastole that contribute to energy loss and reducing systemic flow and worsening HF. The re-entering of blood in the LVAD causes a vicious regurgitant flow loop with the consequence that the pump needs to run at a higher speed to maintain the cardiac output and extend the time of shear stress to the blood increasing hemolysis and thrombogenicity (84). Aortic insufficiency can be managed before or after LVAD implantation, according to the severity of regurgitation.

### Pre-LVAD implantation

If the AR is mild, the patient is treated medically. When AR is more than mild, AV needs to be tackled before or at the time of device implantation to prevent its progression. The intervention of choice is AV replacement with a bio-prosthesis as described below. If the patient already has an AV replaced with a mechanical valve at the time of device implantation, it is recommended to replace it with a bioprosthetic valve, or bypass the valve from the circulation to avoid thromboembolic complications (74, 85). To the best of our knowledge, there are no studies or case report reporting the experience of transcutaneous aortic valve implantation (TAVI) in pre-LVAD patients. This is most likely because TAVI in AR is an off-label procedure, as explained later, and standard aortic valve replacement (SAVR) is often the best option.

### Post-LVAD implantation

Patients with moderate to severe AR after device implantation should be promptly treated since it affects the patient either clinically or prognostically as previously explained (Figure 3) (12–15, 60). When approaching a patient with AR, it is important to determine whether he/she is asymptomatic or symptomatic. When an invasive approach is needed, the options include AV replacement with a bioprosthetic valve by SAVR or permanent AV closure or TAVI.

#### Asymptomatic patient

Management indications are still unclear for patients developing AR on LVAD. Since there is a clear association between the development of AR secondary to permanently closed AV, it is suggested to adjust the LVAD settings to lower pump speed to obtain intermittent AV opening and reducing the risk of *de novo* AR or AR progression (12). On the other hand, it is important to keep in mind that once the LVAD pump speed is reduced other potential complications, such as HF symptoms, increasing LV dimensions, increase MR severity, and LV filling pressure can occur.

Medical management, as described below, can help in AR improvement.

#### Symptomatic patient

In this case, there are two options, non-surgical and surgical.

##### Non-surgical management

1. Medical management can be a starting option, targeting fluid overload with diuretics, reducing afterload with vasodilators (12) along with utilizing angiotensin-converting enzyme inhibitors, calcium channel blockers, or beta blockers (86).
2. Device settings can be adjusted to the lowest speed for intermittent AV opening and improved best functional class (20) but is rarely sufficient (86). Intermittent low-speed algorithm has been developed to simulate normal physiological conditions and AV valve movement, reducing the likelihood of *de novo* AR or AR progression (43, 87).

##### Surgical management

###### During/post-LVAD AV management

If LVAD is expected to be implanted for more than 1 year (12), different AV procedures are performed for different AV pathologies. For degenerative disease (cusp prolapse or malcoaptation) the closure of the AV can be considered while the replacement with a bioprosthetic valve is recommended for calcified valves (12, 88). Bioprosthetic valve is considered to be favorable, however, due to a longer cardioplegic arrest, there is the potential for thromboembolic complications of the prosthesis (89–91), along with increased operative risk and a fivefold increase in 30-day mortality (92). Replacement with a mechanical valve is contraindicated because of the high risk of thrombosis (93). AV closure is contraindicated in patients with a plan of temporary LVAD since it leaves the patients completely dependent on the device and can lead to disastrous complications in those developing pump thrombosis or malfunction (12). Important to note is that patients with AV procedure and LVAD implantation at the same time have higher peri-operative and early mortality, but similar long-term outcomes to patients without simultaneous procedures (94). Furthermore, a recent minimally invasive technique of LVAD implantation with concomitant transapical transcatheter aortic valve replacement through a hybrid process has shown good early outcomes as it preserves the pericardial geometry and eliminates the need for cardioplegic arrest (95).

Importantly, AR can re-develop within the first year in up to 20% of patients having simultaneous AV procedures at the time of LVAD implantation (19% with AV repair, 5% for AV closure, and 9% after AV replacement) (94).

#### Percutaneous management

Prohibitive surgical risk patients can be candidates for percutaneous techniques including percutaneous occluding devices (PODs) or transcatheter aortic valve implantation (TAVI) (96–99). Although TAVI technique at the moment is considered the last line of treatment due to significant complications including mortality, it does look promising and will probably become the first choice of treatment in the near future. Moreover, research effort is put in developing new transcatheter heart valves (THV) dedicated specifically to AR (100, 101). The case of TAVI in AR, is an off-label procedure given that a non-calcific AR is the most common characteristic. In this case AR can be associated with aortic dilatation, aortic annulus enlargement in association with advanced LV and/or RV dysfunction and remodeling. Schneeberger et al. (102), used a self-expanding THV in 9 patients reporting no mortality at 30 days, two acute kidney injury and 2 patients with mild paravalvular leak. Belkin et al. (103) had a more complicated experience. Seven patients underwent TAVI: 2 patients had two procedures; 2 patients had an inadequate fixation with severe paravalvular leak that evolved into cardiogenic shock and death within the first day. In the rest of the 5 patients with successful valve deployment, AR improved significantly. Residual mild or moderate paravalvular leak was noted on five of the six surviving patients on immediate post-procedure TTE as well as at 6-month follow-up. Phan et al. (96) identified 29 patients from 2,116 electronic database search. Eight patients underwent TAVI and 21 POD. The results were similar in terms of AR improvement after the procedure. The POD group was complicated by migration of the device in 2 patients, TAVI group was complicated by device migration in 2 patients and 1 had significant post-implant paravalvular leak. The survival of patients with TAVI at 20 months was 35% while no patient survived beyond 20 months in POD group. A recent study on 148 hospitalized patients with a history of LVAD, compared the outcome of those who underwent TAVI or SAVR (87 TAVR vs. 61 SAVR). The 30-day all-cause readmission rate was numerically higher in the SAVR group, but the difference was not statistically significant, as well as the difference in mortality (104).

**FIGURE 6:**
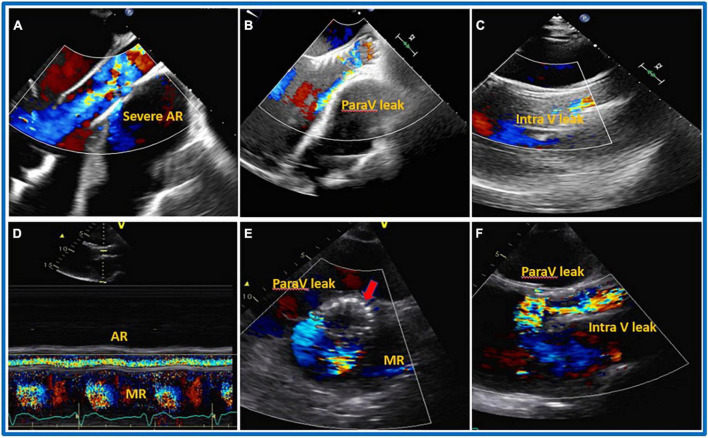
Transcatheter aortic valve implantation (TAVI) procedure. 32 years old gentleman with severe dilated cardiomyopathy, severe reduced systolic function (Ejection Fraction less than 10%), severe dilated and reduce systolic function of the right ventricle (RV), on left ventricular assist device (LVAD) as Bridge To Therapy (BTT) developed severe aortic regurgitation (AR). On transthoracic (TTE) the patients also had severely dilated RV, severe tricuspid regurgitation (TR) and associated severe liver and kidney dysfunction on hemodialysis. The patient underwent TAVI procedure with mild to moderate residual intravalvular AR. Five days after the procedure, TTE was repeated showing the presence of severe PV leak and the possible protrusion of the prosthesis into left ventricular outflow tract (LVOT). The patient died after 10 days from the procedure due to end stage heart failure and multi organ failure. **(A)** Pre-TAVI implantation, **(B)** TTE, mild to moderate paravalvular leak during TAVI procedure and before aortic valve (AV) deployment, **(C)** parasternal long axis TTE just after deployment, mild to moderate intra and paravalvular leak, **(D–F)** 5 days later S/P TAVR with likely severe intra and paravalvular leak, possible protrusion of the AV prosthesis into LV. **(D)** Color M-mode with AR in systole and diastole. **(E)** Shot axis. **(F)** Parasternal long axis.

The most common complications associated with TAVI procedures are embolization of the device into the aorta or migration of the prosthesis in the left ventricle (due to the absence of calcium for stabilizing the valve and the vacuum effect of the inflow cannula), as well as the intra-and paravalvular leaks that either occurs after the valve is released or develop during the follow-up period and call for additional intervention (Figure 6). Prior to TAVI, right ventricular dysfunction, dilatation, and pulmonary hypertension—even those that are not clinically evident—must be carefully assessed. Even though there is not enough data in literature, it is required to use medications like milrinone, nitric oxide, and phosphodiesterase inhibitors prior to the procedure along with carefully managing fluid overload during the procedure to lower the risk of RV failure following TAVI. The main causes of periprocedural death are extensive, widespread organ damage and RV failure (kidney, liver) (105). Table 6 summarizes all the different management options with advantages and disadvantages.

**TABLE 6:** Managing aortic regurgitation (AR) complication that develops post-left ventricular assist device (LVAD) transplantation (12, 89–92).

Management	Medical therapy	Device settings	Bioprosthetic valve	Mechanical valve	AV closure–Felt strips anchored to aortic wall	AV closure–Suture closure of AV commissures	TAVI
Advantages	Reduces systemic afterload and preload.	Simulated normal physiological opening of AV.	Recommended for calcified valves.	Immediate relief.	Tolerable and safe.	Easy, rapid, and 1 year durability in case reports. Reduced possibility of complications.	Symptom relief is immediate. Minimally invasive.
Disadvantages	Allows symptom control and temporary respite.	Rarely adequate.	Longer cardioplegic arrest, potential for thromboembolic complications of the prosthetic valve along with increased operative risk and a fivefold increase in 30-day mortality.	High risk of thrombus development and later embolization.	Hemodynamic instability may result from device malfunction.	Hemodynamic instability may result from device malfunction.	Paravalvular and intravalvular regurgitation. Migration of the device Long-term data are lacking.
Comments	May have cardiogenic shock or be medically resistant to treatment for heart failure, both of which necessitate surgical intervention.	–	Considered to be favorable.	Not advised.	Definitive method.	The technique is fast and reliable.	Possible RV failure due to sudden increased in pre-load.

## Conclusion

The ability of LVAD support to function as a BTT, BTC, and DT has greatly increased its appeal in recent years. A major concern with LVAD implantation is the development of *de novo* AR, which is estimated to have a prevalence of 10–55% in the first 6 months, and the worsening of pre-existing AR. The AV is considerably impacted by the LVAD; hence, this factor should be carefully considered along with the aortic dimension. The specific hemodynamics of AR make it difficult for TTE/TEE to accurately identify this condition, which is crucial because AR affects mortality and morbidity. It is advised that AR be addressed at the time of LVAD implantation if it is moderate or worse before the implantation. Intervention must be taken into consideration if moderate or worse AR occurs after implantation. Non-invasive, medical, and/or LVAD pump modification settings, or invasive management options are available. Since patients frequently have advanced HF with severely compromised end organ damage and high-risk surgery, TAVI is regarded as a possible alternative to invasive therapies, even though it is currently off-label.

## Author contributions

OV, AM, AS, and AE-S equally contributed in writing the manuscript and editing. KF, AE, HA, NK, MA, DA-B, ARS, and FT assisted in visualization and editing. All authors contributed to the article and approved the submitted version.
